# Nonpharmaceutical Measures for Pandemic Influenza in Nonhealthcare Settings—International Travel-Related Measures

**DOI:** 10.3201/eid2605.190993

**Published:** 2020-05

**Authors:** Sukhyun Ryu, Huizhi Gao, Jessica Y. Wong, Eunice Y.C. Shiu, Jingyi Xiao, Min Whui Fong, Benjamin J. Cowling

**Affiliations:** University of Hong Kong, Hong Kong, China (S. Ryu, H. Gao, J.Y. Wong, E.Y.C. Shiu, J. Xiao, M.W. Fong, B.J. Cowling);; Konyang University, Daejeon, South Korea (S. Ryu)

**Keywords:** influenza, pandemic, viruses, respiratory diseases, nonpharmaceutical interventions, entry screening, border closure, travel restrictions, quarantine, vaccine-preventable diseases, public health

## Abstract

International travel–related nonpharmaceutical interventions (NPIs), which can include traveler screening, travel restrictions, and border closures, often are included in national influenza pandemic preparedness plans. We performed systematic reviews to identify evidence for their effectiveness. We found 15 studies in total. Some studies reported that NPIs could delay the introduction of influenza virus. However, no available evidence indicated that screening of inbound travelers would have a substantial effect on preventing spread of pandemic influenza, and no studies examining exit screening were found. Some studies reported that travel restrictions could delay the start of local transmission and slow international spread, and 1 study indicated that small Pacific islands were able to prevent importation of pandemic influenza during 1918–19 through complete border closure. This limited evidence base indicates that international travel-related NPIs would have limited effectiveness in controlling pandemic influenza and that these measures require considerable resources to implement.

From time to time, novel influenza A virus strains emerge and cause global influenza pandemics (*1*). Pandemics occurred 3 times in the 20th century and 1 time so far in the 21st century (*2*). The recognition that influenza pandemics can have substantial social and economic effects in addition to the impact on public health, along with the emergence of highly pathogenic strains of avian influenza virus in the past 20 years, has stimulated greater attention in preparing for future influenza pandemics (*3*,*4*). Given the delays in the availability of specific vaccines and limited supplies of antiviral drugs, nonpharmaceutical interventions (NPIs) form a major part of pandemic plans (*2*). 

A range of NPIs can be applied at international, national, and local levels, with the objectives of delaying the arrival of infected persons, slowing the spread of infection, delaying the epidemic peak, and reducing the size of the peak (*5*). This article focuses on the use of measures related to international travel, including entry and exit screening of travelers for infection, travel restrictions, and border closures (Table 1). We aimed to review the evidence base assessing the effectiveness of these travel-related NPIs against pandemic influenza and to identify the barriers to implementation of these interventions.

**Table 1:** Terminology of international travel-related nonpharmaceutical interventions

Screening travelers	International travel restriction	Border closure
Screening travelers entering or leaving a country for signs and symptoms of influenza virus infection or recent exposure to influenza virus infection by using health declaration forms, visual inspections, thermal scanners, or any combination of these measures (*6*)	Limitations on travel between particular countries (*7*)	Complete prevention of movement of individuals into and out of a particular country (*7*)

## Methods and Results

We searched for literature reporting or estimating the effectiveness of NPIs related to international travel and movement, including entry and exit screening travelers, travel restrictions, and border closures on pandemic or interpandemic influenza. We conducted literature searches on PubMed, Medline, Embase, and Cochrane Library for peer-reviewed articles published from January 1, 1946, through April 28, 2019. The search terms used were identified from relevant systematic reviews and research reports (*8*,*9*). We collected additional studies from secondary references from included studies or other relevant searches. Articles were eligible for inclusion if they reported or estimated the effectiveness of international travel–related NPIs for pandemic influenza using quantitative indicators such as delaying the introduction of infection, delaying the epidemic peak, or reducing the size of the peak. We excluded articles if they did not investigate the quantitative effectiveness of international travel–related NPIs or were editorials, reviews, or commentaries without primary data. Furthermore, we restricted articles to those published in English. Two independent reviewers (S.R. and H.G.) screened titles and abstracts and assessed full-text articles for eligibility. A third reviewer (B.J.C.) adjudicated any disagreements between the 2 reviewers.

We extracted the information on the effectiveness of NPIs from included studies by using a structured data-extraction form. Information of interest included the study setting, specific measures implemented, timing of intervention implementation, study results regarding effectiveness indicators, and potential barriers to implementation. The assessment of quality of evidence considered study design and assigned generally higher quality to randomized trials, lower quality to observational studies, and lowest quality to simulation studies. We provide full search terms, search strategies, selection of articles, and summaries of the selected articles (Appendix).

### Screening Travelers for Infection

We identified 4 relevant studies that considered the effect of screening on influenza transmission, including 2 epidemiologic studies from the 2009 pandemic (*10*,*11*) and 2 simulation studies (*12*,*13*). The epidemiologic studies estimated that entry screening delayed the arrival of influenza A(H1N1)pdm09 virus to previously unaffected areas by an average of 7–12 days (*11*) and delayed the epidemic in China by 4 days by reducing imported cases by 37% from border entry screening (*10*). The simulation studies predicted that entry screening would delay the arrival of infection into a country by a few days or 1–2 weeks at most (*12*,*13*). We did not identify any studies on exit screening; in the 2009 influenza pandemic, exit screening was not implemented by Mexico (*14*), nor by most other countries.

We did not systematically review studies of the technical performance of various screening tools (e.g., screening case definitions and thermal scanners) but identified in an informal search 4 studies that discussed the challenges of screening travelers for infection, which include limited screening sensitivity (*10*,*11*,*13*), an incubation period of 1–7 days for influenza A(H1N1)pdm09 virus (meaning some infected travelers might not show symptoms until after arrival at their destination) (*10*,*12*,*13*), limited local capacity of influenza surveillance (*10*,*11*), and limited public health resources, such as laboratory capacity and funding (*10*,*11*,*13*).

Screening inbound travelers for infection is a very visible public health intervention and can reduce the number of infectious persons entering the country (*15*). Infrared thermometers are currently used in many ports of entry in Asia because of the instantaneous and noninvasive nature of their use. Several simulation studies (*10*–*13*) included in this review estimated that this intervention helped to delay the introduction of infected persons. However, the sensitivity of screening travelers has been largely reliant on the sensitivity of detection of fever. Epidemiologic studies (*16*,*17*) conducted during the 2009 influenza pandemic demonstrated the low detection rate of entry screening that used the infrared thermal scanner and health declaration form at the airport; the sensitivity of screening travelers for infection was 5.8% in New Zealand and 6.6% in Japan. In addition to the lack of sensitivity for detecting febrile travelers (e.g., some travelers with febrile illness might take antipyretic medicine and evade detection), some infected travelers might travel during the incubation period, which is typically 1–2 days, and thus would not be identified as infected at departure or arrival (*10*,*12*). Once infection begins spreading in a local community, identifying additional inbound travelers with infection will do little to limit local spread. In addition, entry screening consumes considerable public health resources, including trained staff, screening devices, and laboratory resources, and thus might not be justifiable (*18*).

### Travel Restrictions

We identified 1 epidemiologic study and 9 simulation studies that estimated or predicted the effectiveness of international travel restrictions (*19*–*28*) (Table 2). An epidemiologic study estimated that the peak in the number of influenza-associated deaths was delayed by 2 weeks when international flight volume was reduced by 27% (*2*8). Simulation studies predicted that 90%–99% of travel restrictions could delay international spread of cases by 2–19 weeks (*20*), delay the importation of the first case-patients by 1–8 weeks (*23*–*26*), and delay the epidemic peak by 1–12 weeks (*19*,*23*,*24*,*26*,*27*).

**Table 2:** Overall summary of effectiveness international travel-related non-pharmaceutical interventions for reducing influenza transmission

Objective	Screening travelers	Travel restriction	Border closure
Delaying introduction of case	• Likely delay by 4 d with detection rate of 37% travelers identified from the port of entry at the border (*10*)* • Associated with mean additional delay of case importation (7–12 d, 95% CI 0–30days, 2009 H1N1 pandemic) (*11*)* • Might delay 3 d reaching 20 infected cases at risk-country (R_0_ = 1.5 with 400 travelers/day) (*12*) • Might delay importation of infected case-patientss (21–1555 d, 2009 H1N1 pandemic) (*13*)	• The mean time delays for exporting the infected case is 5.3 d (80% restriction), 11.7 d (90%), and 131.7 d (99%) (R_0_ = 1.8 with implementation of 20 d from first case occurred) (*20*)* • Among 17 Pacific Island countries and territories, with 99% restriction, 6 countries (with R_0_ = 1.5) and 4–5 countries (with R_0_ >2.25) would likely escape the pandemic influenza with >50% probability (implemented at very beginning of pandemic) (*21*) • Full children-selective travel restriction might delay an epidemic by 19–35 d (R_0_ = 1.2), and less than 15 d (R_0_ = 1.6 and 2.0, implemented after pandemic declared) (*22*) • Mean delay of the first imported case in influenza-unaffected countries was estimated <3 d (40% restriction), and ≈2 weeks (90% restriction) with R_0_ = 1.7 and implementation after pandemic declared (*23*) • Likely delay interval between first global case and the importation of the first cases by 7–37 d (R_0_ = 1.4, 1.7, or 2; 90% or 99% restriction; implemented 30 d after first global case occurrence) (*24*) • Might delay the first passage time of infected case-patient from 18 d to 31 d (outbreak originated from Hong Kong) and from 7 d to 27 d (from Sydney) with R_0_ = 1.7 (*25*) • A 99% restriction of air-only, both air and land, and all modes of transportation might delay the interval between the first imported case and 100 infected case-patients passed the border by a week, 1–2 weeks, and 2 mo, respectively (R_0_ = 1.4; implemented on the day after the first global case reported) (*26*)	• Arrival of influenza pandemic was significantly delayed and reduced compare with the other Pacific Island Jurisdictions (*29*)*
Delaying the epidemic peak	• Not available	• Imported infections might delay the epidemic peak of the United States by 1.5 wks (90% restriction), 3 wks (99%), or 6 wks (99.9%) with R_0_ = 1.4–2.0 (implemented 30 d into global pandemic) (*19*) • Might delay pandemic peak by 6–39 d (R_0_ = 1.4, 1.7, or 2; 90% or 99% restriction; implemented 30 d after first global case occurrence) (*24*) • Might delay epidemic peak by 2 wks (99% air travel restriction), 3.5 wks (99% air and land travel restriction), and 12 wks (99% all mode of transportation) with R_0_ = 1.4 (*26*) • Might delay median epidemic peak by 7–102 d (R_0_ = 1.8–5; 50%–99.9% restriction) (*27*) • Peak of influenza mortality delayed by 2 wks (27% international flight volume reduction) (*28*)	• Not available
Reducing the size of the peak	• Not available	• Not available	• Not available

*Epidemiology study.

A simulation study predicted that selectively restricting the travel of children could delay the spread of infection by 35 days (R_0_ = 1.2–2.0) (*22*), and another simulation study assessing the probability of escaping 1918–19 influenza pandemic among 17 Pacific Island countries and territories estimated that 4–5 countries avoided influenza pandemic (R_0_ = 1.5–3.0) by strict limitation (79% or 99% restriction) of incoming travelers (*21*). Three studies explored the barriers to travel restrictions, which included the threat of economic loss (*21*,*26*) and lack of compliance among the public (*20*).

Because the volume of transportation is associated with the spread of influenza (*28*,*30*), travel restrictions have been considered as a measure to reduce international spread (*31*). Although previous expert survey and reviews suggested that travel restrictions are less likely to be effective (*8*,*9*,*32*), international travel restrictions are still included in some national pandemic plans (*33*). Several of the studies we reviewed (*19*,*20*,*22*–*28*) predicted that international travel restrictions might delay the importation of new infected persons from other affected areas, slow the international spread of the epidemic, and delay the epidemic peak (*25*). However, simulation studies estimated that travel restrictions after 5 months of the international arrival of the first infected persons would not be effective (*2*6) and that only strict travel restriction was likely to be effective (*19*); thus, the time of implementation of this measure should be considered with strict travel restrictions at the early stage of a pandemic. Some barriers exist to implementation of travel restrictions against pandemic influenza, most notably the potential economic consequences of restricting business travelers, as well as legal and ethical issues regarding mobility restrictions (*34*), discrimination of persons from influenza-affected area (*35*), and lack of public compliance.

### Border Closures

One study investigated the effectiveness of border closures in 11 South Pacific Island jurisdictions during the 1918–19 influenza pandemic. We identified 4 islands where strict border control, including 5–7 days of maritime quarantine, substantially delayed the importation of influenza from 3 to 30 months and reduced the mortality rate compared with the other islands that had not implemented border control (*36*).

Because travel can drive cross-border transmission of infectious diseases, complete border closure could in theory prevent or delay the spread of influenza or its introduction in previously unaffected countries (*21*,*36*). However, in practice, complete border closure is likely to be unfeasible, even on isolated islands, because of the need to import food and medical supplies (*21*), and would result in substantial economic and social disruption (*34*).

## Discussion

We reviewed the effectiveness of each international travel–related NPI and the barriers to its implementation to provide scientific evidence to public health authorities. Our review found that the effect of screening travelers on entry to a country or region is very limited and unlikely to be a rational use of resources. However, this intervention has a potential role to inform travelers about the risk for infection and provide travel advice on avoiding travel to certain regions after departure or how to seek treatment after arrival (*16*). Furthermore, such screening can be seen by policy makers and politicians as a visible public health measure to help assure the public that action is being taken (*16*).

Our review identified the potential threat of economic consequences as a major barrier to implementation of travel restrictions. A simulation study demonstrated that children-selective travel restriction during a pandemic is less likely to affect economic impact compared with nonselective travel restrictions (*22*). A more structured epidemiologic study is needed to examine the cost and benefit of travel restriction by different risk groups of influenza transmission. A previous study demonstrated that successful border closure for 6 months in an island country provided a net societal benefit of USD 7.3 billion (*36*). However, this extreme measure is unlikely to be implemented unless required by national law in extraordinary circumstances during a very severe pandemic. The literature on border closure included in our review was based on the historical scenario of the 1918–19 influenza pandemic in isolated islands; this research might have limited relevance given the current and ever increasing levels of globalization.

Although international travel–related NPIs are not likely to be able to prevent importation of pandemic influenza to a country or region, NPIs implemented at the early phase might delay the start of a local epidemic by a few days or weeks (*37*), which is important if such delay can contribute to reducing the effect of the epidemic (e.g., by buying time to prepare healthcare providers and the public before the arrival of the epidemic, to plan and coordinate social distancing measures, and to purchase additional pharmaceuticals such as antiviral drugs or vaccines) (*38*). Once an epidemic has started, travel restrictions might also be used to delay the peak of the epidemic in an isolated location where heavy seeding by incoming infected persons could accelerate local transmission. International Health Regulations could play a role in decisions on whether to implement certain international measures (*39*).

We identified several knowledge gaps that could be filled by further research. Most fundamentally, information is still lacking on some aspects of the basic epidemiology of influenza, including the dynamics of person-to-person transmission (e.g., Can a person be infectious before the onset of symptoms? Can transmission occur from an asymptomatic or pauci-symptomatic case-patient? What fraction of infections are asymptomatic?). In terms of specific research on the effectiveness of travel-related NPIs, it is difficult to envisage how intervention studies could be done, but epidemiologic studies could be planned in advance of influenza pandemics or perhaps severe influenza epidemics. Studies could answer questions such as how many infections are imported from overseas or whether travel advisories might encourage infected persons not to travel.

Our review needs to be interpreted in light of some limitations. First, although international travel or trade of infected animals might have a role in the international spread of influenza, the study that assessed the movement restriction of animals was not included in this review. Second, mathematical models are useful tools for investigating the advantages and disadvantages of different interventions, but the results often depend on key modeling assumptions that are difficult to verify (*19*). The assessment of the quality of evidence was considered weak overall, given that most of the epidemiologic studies included in our review were ecologic studies. Third, only a few studies on the ethical and economic considerations regarding travel-related measures during influenza epidemics and pandemics were available (*26*,*40*).

Many countries continue to update their influenza pandemic plans on the basis of the latest available evidence. We found that international travel–related NPIs could delay the introduction of influenza and delay the start of local transmission; however, limited evidence exists to inform the use of these NPIs for controlling pandemic influenza. The evidence that we identified in our review does not support entry screening as an efficient or effective measure, and travel restrictions and border closures are likely to be too disruptive to consider. Additional prospective research on the effectiveness of travel-related NPIs would be valuable to support evidence-based decisions for future influenza pandemics.

Appendix Additional information about nonpharmaceutical measures for pandemic influenza in nonhealthcare settings—international travel-related measures.

## References

[1] Dawood FS, Jain S, Finelli L, Shaw MW, Lindstrom S, Garten RJ, et al.; Novel Swine-Origin Influenza A (H1N1) Virus Investigation Team. Emergence of a novel swine-origin influenza A (H1N1) virus in humans. N Engl J Med. 2009;360:2605–15. 10.1056/NEJMoa0903810 19423869

[2] The Lancet Infectious Diseases. The Lancet Infectious Diseases. How to be ready for the next influenza pandemic. Lancet Infect Dis. 2018;18:697. 10.1016/S1473-3099(18)30364-5 29976515

[3] Smith RD, Keogh-Brown MR, Barnett T, Tait J. The economy-wide impact of pandemic influenza on the UK: a computable general equilibrium modelling experiment. BMJ. 2009;339(nov19 1):b4571. 10.1136/bmj.b4571 PMC2779854 19926697

[4] World Health Organization. Draft thirteenth general programme of work, 2019–2023. 2018 [cited 2019 Jul 10]. http://apps.who.int/gb/ebwha/pdf_files/WHA71/A71_4-en.pdf

[5] Bell D, Nicoll A, Fukuda K, Horby P, Monto A, Hayden F, et al.; World Health Organization Writing Group. Non-pharmaceutical interventions for pandemic influenza, national and community measures. Emerg Infect Dis. 2006;12:88–94. 16494723 10.3201/eid1201.051371 PMC3291415

[6] European Centre for Disease Prevention and Control. Infection prevention and control measures for Ebola virus disease: entry and exit screening measures. 2014 [cited 2019 Jul 10]. https://www.ecdc.europa.eu/en/publications-data/infection-prevention-and-control-measures-ebola-virus-disease-entry-and-exit

[7] US Homeland Security Council. National strategy for pandemic influenza implementation plan. 2006 [cited 2019 Jul 10]. https://www.cdc.gov/flu/pandemic-resources/pdf/pandemic-influenza-implementation.pdf

[8] Mateus AL, Otete HE, Beck CR, Dolan GP, Nguyen-Van-Tam JS. Effectiveness of travel restrictions in the rapid containment of human influenza: a systematic review. Bull World Health Organ. 2014;92:868–880D. 10.2471/BLT.14.135590 25552771 PMC4264390

[9] Lee VJ, Lye DC, Wilder-Smith A. Combination strategies for pandemic influenza response - a systematic review of mathematical modeling studies. BMC Med. 2009;7:76. 10.1186/1741-7015-7-76 20003249 PMC2797001

[10] Yu H, Cauchemez S, Donnelly CA, Zhou L, Feng L, Xiang N, et al. Transmission dynamics, border entry screening, and school holidays during the 2009 influenza A (H1N1) pandemic, China. Emerg Infect Dis. 2012;18:758–66. 10.3201/eid1805.110356 22515989 PMC3358060

[11] Wu JT, Cowling BJ, Lau EH, Ip DK, Ho LM, Tsang T, et al. School closure and mitigation of pandemic (H1N1) 2009, Hong Kong. Emerg Infect Dis. 2010;16:538–41. 10.3201/eid1603.091216 20202441 PMC3206396

[12] Caley P, Becker NG, Philp DJ. The waiting time for inter-country spread of pandemic influenza. PLoS One. 2007;2:e143. 10.1371/journal.pone.0000143 17206278 PMC1764036

[13] Malone JD, Brigantic R, Muller GA, Gadgil A, Delp W, McMahon BH, et al. U.S. airport entry screening in response to pandemic influenza: modeling and analysis. Travel Med Infect Dis. 2009;7:181–91. 10.1016/j.tmaid.2009.02.006 19717097 PMC7185379

[14] Khan K, Eckhardt R, Brownstein JS, Naqvi R, Hu W, Kossowsky D, et al. Entry and exit screening of airline travellers during the A(H1N1) 2009 pandemic: a retrospective evaluation. Bull World Health Organ. 2013;91:368–76. 10.2471/BLT.12.114777 23678200 PMC3646347

[15] Read JM, Diggle PJ, Chirombo J, Solomon T, Baylis M. Effectiveness of screening for Ebola at airports. Lancet. 2015;385:23–4. 10.1016/S0140-6736(14)61894-8 25467590

[16] Hale MJ, Hoskins RS, Baker MG. Screening for influenza A(H1N1)pdm09, Auckland International Airport, New Zealand. Emerg Infect Dis. 2012;18:866–8. 10.3201/eid1805.111080 22516105 PMC3358051

[17] Sakaguchi H, Tsunoda M, Wada K, Ohta H, Kawashima M, Yoshino Y, et al. Assessment of border control measures and community containment measures used in Japan during the early stages of Pandemic (H1N1) 2009. PLoS One. 2012;7:e31289. 10.1371/journal.pone.0031289 22355354 PMC3280294

[18] Priest PC, Jennings LC, Duncan AR, Brunton CR, Baker MG. Effectiveness of border screening for detecting influenza in arriving airline travelers. Am J Public Health. 2013;103:1412–8. 10.2105/AJPH.2012.300761 23237174 PMC4007855

[19] Ferguson NM, Cummings DA, Fraser C, Cajka JC, Cooley PC, Burke DS. Strategies for mitigating an influenza pandemic. Nature. 2006;442:448–52. 10.1038/nature04795 16642006 PMC7095311

[20] Hollingsworth TD, Ferguson NM, Anderson RM. Will travel restrictions control the international spread of pandemic influenza? Nat Med. 2006;12:497–9. 10.1038/nm0506-497 16675989

[21] Eichner M, Schwehm M, Wilson N, Baker MG. Small islands and pandemic influenza: potential benefits and limitations of travel volume reduction as a border control measure. BMC Infect Dis. 2009;9:160. 10.1186/1471-2334-9-160 19788751 PMC2761921

[22] Lam EH, Cowling BJ, Cook AR, Wong JY, Lau MS, Nishiura H. The feasibility of age-specific travel restrictions during influenza pandemics. Theor Biol Med Model. 2011;8:44. 10.1186/1742-4682-8-44 22078655 PMC3278369

[23] Bajardi P, Poletto C, Ramasco JJ, Tizzoni M, Colizza V, Vespignani A. Human mobility networks, travel restrictions, and the global spread of 2009 H1N1 pandemic. PLoS One. 2011;6:e16591. 10.1371/journal.pone.0016591 21304943 PMC3031602

[24] Ciofi degli Atti ML, Merler S, Rizzo C, Ajelli M, Massari M, Manfredi P, et al. Mitigation measures for pandemic influenza in Italy: an individual based model considering different scenarios. PLoS One. 2008;3:e1790. 10.1371/journal.pone.0001790 18335060 PMC2258437

[25] Epstein JM, Goedecke DM, Yu F, Morris RJ, Wagener DK, Bobashev GV. Controlling pandemic flu: the value of international air travel restrictions. PLoS One. 2007;2:e401. 10.1371/journal.pone.0000401 17476323 PMC1855004

[26] Chong KC, Ying Zee BC. Modeling the impact of air, sea, and land travel restrictions supplemented by other interventions on the emergence of a new influenza pandemic virus. BMC Infect Dis. 2012;12:309. 10.1186/1471-2334-12-309 23157818 PMC3577649

[27] Cooper BS, Pitman RJ, Edmunds WJ, Gay NJ. Delaying the international spread of pandemic influenza. PLoS Med. 2006;3:e212. 10.1371/journal.pmed.0030212 16640458 PMC1450020

[28] Brownstein JS, Wolfe CJ, Mandl KD. Empirical evidence for the effect of airline travel on inter-regional influenza spread in the United States. PLoS Med. 2006;3:e401. 10.1371/journal.pmed.0030401 16968115 PMC1564183

[29] McLeod MA, Baker M, Wilson N, Kelly H, Kiedrzynski T, Kool JL. Protective effect of maritime quarantine in South Pacific jurisdictions, 1918-19 influenza pandemic. Emerg Infect Dis. 2008;14:468–70. 10.3201/eid1403.070927 18325264 PMC2570822

[30] Fang LQ, Wang LP, de Vlas SJ, Liang S, Tong SL, Li YL, et al. Distribution and risk factors of 2009 pandemic influenza A (H1N1) in mainland China. Am J Epidemiol. 2012;175:890–7. 10.1093/aje/kwr411 22491083 PMC3339311

[31] Wood JG, Zamani N, MacIntyre CR, Beckert NG. Effects of internal border control on spread of pandemic influenza. Emerg Infect Dis. 2007;13:1038–45. 10.3201/eid1307.060740 18214176 PMC2878213

[32] Aledort JE, Lurie N, Wasserman J, Bozzette SA. Non-pharmaceutical public health interventions for pandemic influenza: an evaluation of the evidence base. BMC Public Health. 2007;7:208. 10.1186/1471-2458-7-208 17697389 PMC2040158

[33] World Health Organization. Comparative analysis of national pandemic influenza preparedness plans. 2011 [cited 2019 Jul 10]. https://www.who.int/influenza/resources/documents/comparative_analysis_php_2011_en.pdf

[34] World Health Organization. Ethical consideration in developing a public health response to pandemic influenza. 2007 [cited 2019 Jul 10]. https://www.who.int/csr/resources/publications/WHO_CDS_EPR_GIP_2007_2c.pdf

[35] World Health Organization. Guidance for managing ethical issues in infectious disease outbreaks. 2016 [cited 2019 Jul 10]. http://www.who.int/iris/handle/10665/250580

[36] Boyd M, Baker MG, Mansoor OD, Kvizhinadze G, Wilson N. Protecting an island nation from extreme pandemic threats: Proof-of-concept around border closure as an intervention. PLoS One. 2017;12:e0178732. 10.1371/journal.pone.0178732 28622344 PMC5473559

[37] Bell D, Nicoll A, Fukuda K, Horby P, Monto A, Hayden F, et al.; World Health Organization Writing Group. Non-pharmaceutical interventions for pandemic influenza, international measures. Emerg Infect Dis. 2006;12:81–7. 10.3201/eid1201.051370 16494722 PMC3291414

[38] World Health Organization. Pandemic influenza risk management. 2017 [cited 2019 Jul 10]. https://www.who.int/influenza/preparedness/pandemic/influenza_risk_management

[39] World Health Organization. International Health Regulations (2005), 3rd edition. 2016 [cited 2019 Jul 10]. https://apps.who.int/iris/handle/10665/246107

[40] Saunders-Hastings P, Crispo JAG, Sikora L, Krewski D. Effectiveness of personal protective measures in reducing pandemic influenza transmission: A systematic review and meta-analysis. Epidemics. 2017;20:1–20. 10.1016/j.epidem.2017.04.003 28487207

