# Supplementary material for: Nonpharmaceutical Measures for Pandemic Influenza in Nonhealthcare Settings—International Travel-Related Measures
Source: Emerg Infect Dis. 2020 May;26(5):961–6. doi: 10.3201/eid2605.190993 (PMC7181936; doi:10.3201/eid2605.190993)
Supplement: Appendix — Additional information about nonpharmaceutical measures for pandemic influenza in nonhealthcare settings—international travel-related measures. [file 19-0993-Techapp-s1.pdf]

Article DOI: https://doi.org/eid2605.190993

Nonpharmaceutical Measures for Pandemic Influenza in Nonhealthcare Settings—International Travel–Related Measures

Appendix

1. Screening travelers

1.1 Terminology

Screening travelers for influenza is to reduce the number of infectious individuals entering or leaving a country. Screening measures include health declarations, visual inspection, and thermography to detect individuals with influenza-related symptoms (*1*). These measures can be conducted at arrival terminal (entry screening) or at departure terminal (exit screening) (*2*).

1.2 Search strategy

The databases including PubMed, Medline, EMBASE and Cochrane Library were searched using search terms from 1946 to 28April 2019. Inclusion criteria were primary research evaluating entry and/or exit screening for influenza in the community setting. Studies had to demonstrate any effectiveness following entry and/or exit screening in the community. We excluded studies conducting at the healthcare settings, animal-related studies, systematic reviews and/or meta-analysis without updated evidences, not measuring effectiveness of travel advice to the community, and article type of letter, commentary or news. Two reviewers (SR and HG) contributed to the title, abstract, and full-text screening (Appendix Table 1).

Appendix Table 1. Search strategy for screening travelers

Search terms	Search date	Reviewers
#1. travel or traveler or travelers or traveller or travelers<br>#2. screen or screening or entry screening or exit screening or entry-exit screening or massive screening or boarder screening or detect or detecting or detection<br>#3. influenza or flu<br>#4. #1 and #2 and #3	29 April 2019	SR<br>HG

1.3 Findings

776 records were identified and included in the title and abstract screening, and 741 were excluded. 35 full-texts were evaluated for eligibility and 31 full-texts were excluded. Four full-length articles were included in this systematic review. Flowchart of study selection shown in Appendix Figure 1 and study details are shown in Appendix Table 2.

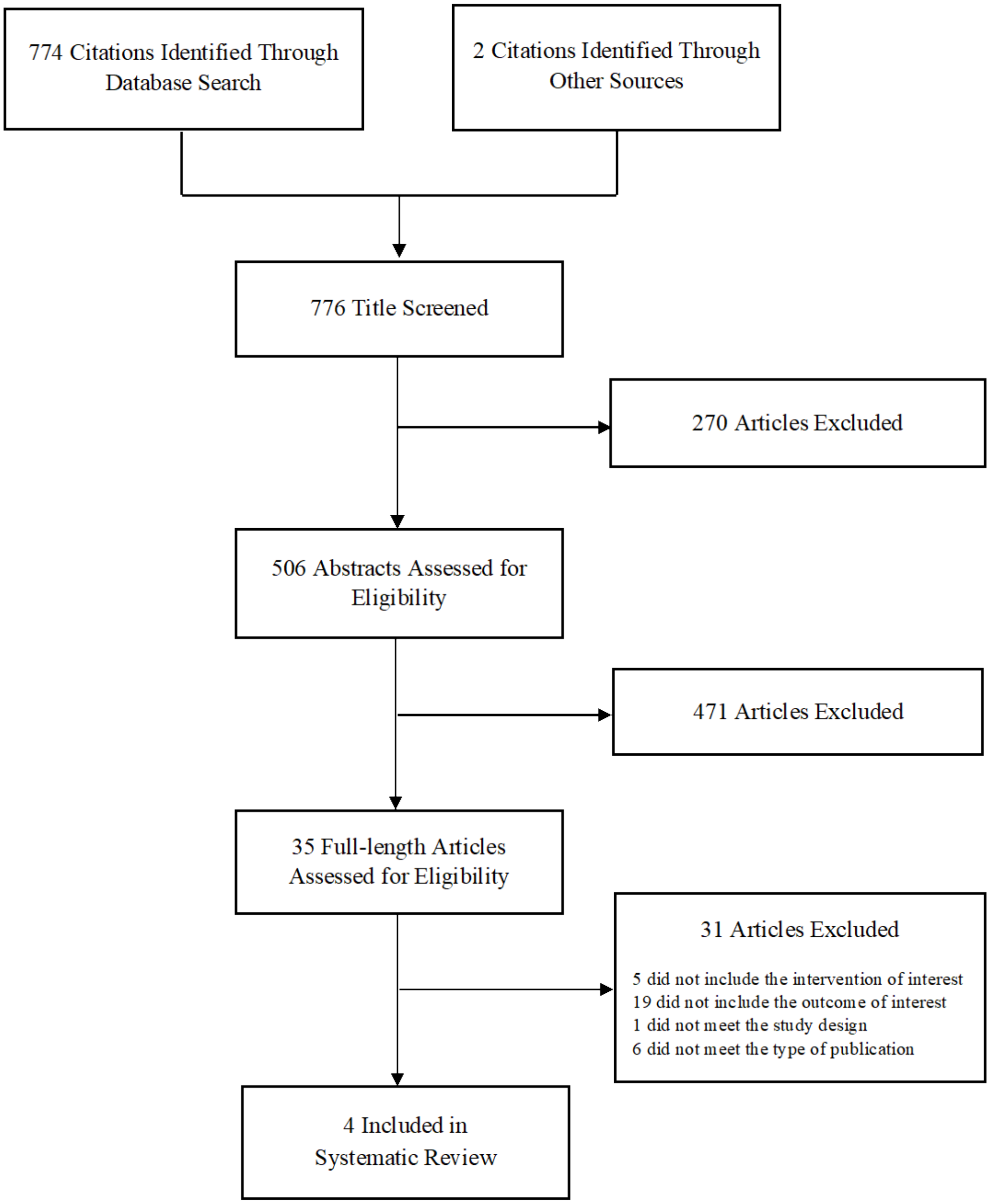

Appendix Figure 1. Flowchart of literature search and study selection for screening travelers.

Appendix Table 2. Summary of studies included in the review of screening travelers

Screening travelers	Study setting	Specific measures (Location and methods)	Timing of implementation	Study results by effective indicators
Caley, et al. (2007) (*3*)	• Simulation of a new influenza pandemic (R_0 = 1.5, 2.5, and 3.5) has emerged and 400 travelers per day tried to depart from the source region where population is 5 million.	• Entry and/or exit screening with 100% sensitivity for identifying cases presenting symptom including rhinorrhea, nasal congestion, sore throat, cough, fever and sense of fever.	• Beginning of influenza pandemic	• The expected median additional delay at- risk country occurred 20 infected cases was increased from 57 to 60 d (R_0 = 1.5 with 400 travelers per a day). However, there was no delay of reaching 20 cases (R_0 = 3.5 with 400 travelers per a day).
Cowling, et al. (2010) (*4*)	• Overall, 35 countries where entry screening policies conducted during 2009 influenza pandemic were reviewed using the official report including first confirmed imported infected case and the first confirmed local infected case or untraceable case.	• Entry screening including medical check before disembarkation, health declaration forms, symptom screening, and thermal scanners.	• Not available	• The entry screening policy was associated with mean additional delay of case importation (7–12 d, 95% CI: 0–30days)
Malone, et al. (2009) (*5*)	• A stochastic discrete simulation of pandemic influenza at U.S. airport entry screening with R_0 = 2.4 from Europe, 2.1 from Latin America, and 2.0 from Canada and U.S..<br>• 50% or 100% infected passengers were screened with <50% or 80% sensitivity of screening.	• Entry screening to detect symptomatic travelers	• Not available	• Delay the importation of the peak of infected cases (21–1555 d)
Yu, et al. (2012) (*6*)	• Case-based surveillance with investigation of cases linked to international travel and entry screening at the Chinese border were conducted during Influenza H1N1 pandemic in 2009, China.	• Entry screening to detect travelers presenting symptoms	• Early of influenza pandemic	• Epidemic is likely delayed by 4 d in China with 37% of infected international travelers identified from the entry screening at the border.

2. International travel restriction

2.1 Terminology

Because the airports, land transportation and maritime transportation are associated with long-distance spread of influenza (*7*), travel restrictions are considered as a measure to reduce regional and international spread (*8*). International travel restriction is to prevent the travel between particular countries (*9*).

2.2 Search strategy

We conducted a search using a search terms in the databases, including PubMed, Medline, EMBASE and Cochrane Library, from 1946 to 28 April 2019. Inclusion criteria were primary

research evaluating international travel restriction for influenza endemic or pandemic in the community setting. Studies had to demonstrate any effectiveness following international travel restriction to the influenza transmission. We excluded studies conducting at the healthcare settings, animal-related studies, systematic reviews and/or meta-analysis without using primary data, not measuring effectiveness of travel restriction to the community, and article type of letter, commentary or news without primary data. Two reviewers (SR and HG) contributed to the title, abstract, and full-text screening (Appendix Table 3).

Appendix Table 3. Search strategy for international travel restriction

Search terms	Search date	Reviewers
#1. travel or traveler or travelers or traveller or travelers<br>#2. international or abroad<br>#3. restrict or restriction or prohibit or prohibition or limit or limitation or control<br>#4. influenza or flu<br>#5. #1 and #2 and #3 and #4	29 April 2019	SR<br>HG

2.3 Findings

554 records were identified and included in the title and abstract screening, and 535 were excluded. Nineteen full-text were evaluated for eligibility and 9 of full-text were excluded. Ten full-length articles were included in this systematic review. The flowchart of study selection is shown in Appendix Figure 2 and the summary of studies is shown in Appendix Table 4.

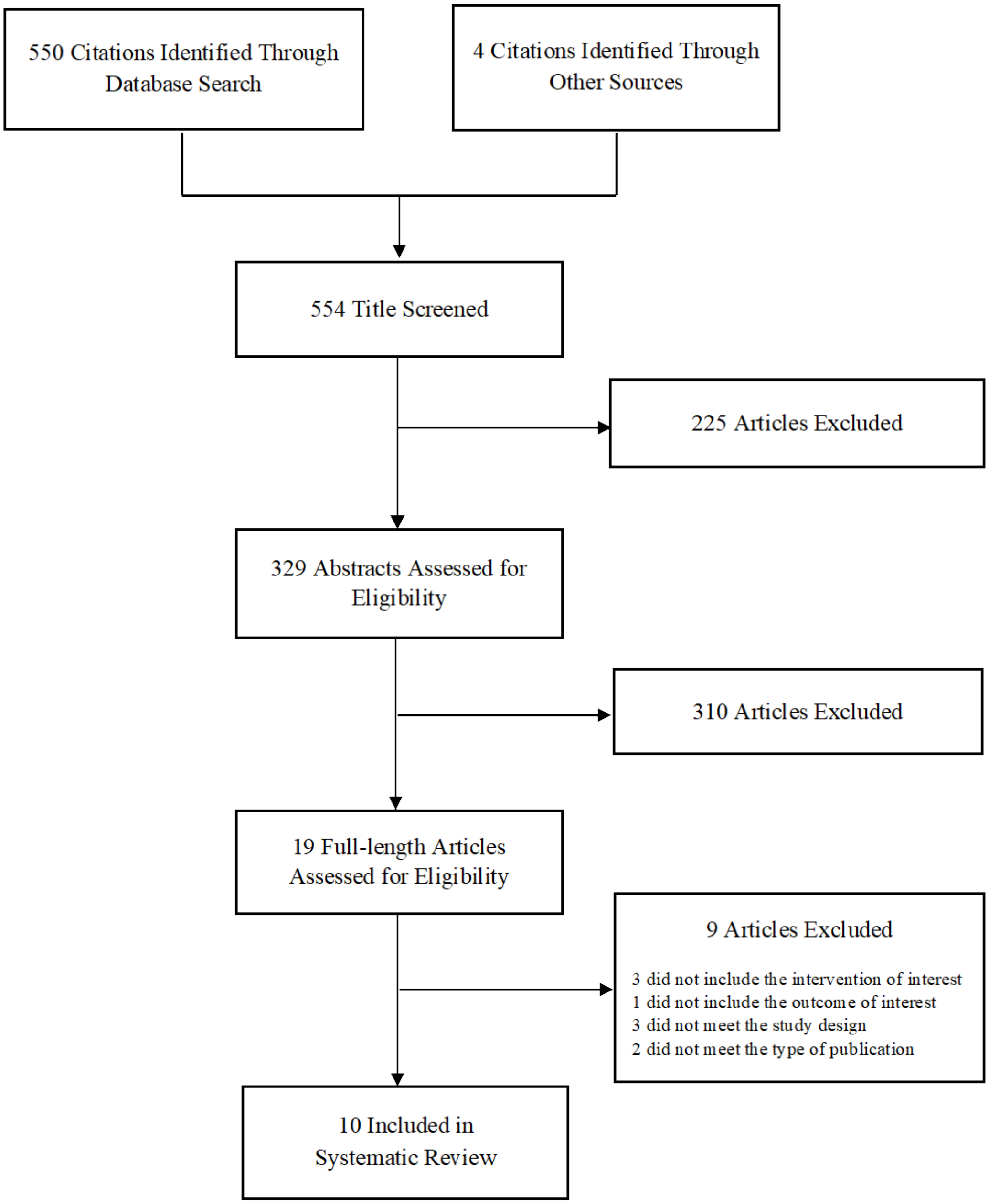

Appendix Figure 2. Flowchart of literature search and study selection for international travel restriction.

Appendix Table 4. Summary of studies included in the review of international travel restriction

International travel restriction	Study setting	Specific measures (Location and methods)	Timing of implementation	Study results by effective indicators
Ciofi degli Atti, et al. (2008) (*10*)	• Global deterministic SEIR model of pandemic influenza (R_0 = 1.4, 1.7, or 2.0) in Italy was conducted.	• Incoming international flight restriction (90 or 99%)	• Thirty days after the first global case occurred	• International air travel restriction may delay the interval between first global case and the importation of the first cases by 7–37 d.<br>• The pandemic peak may delay by 6–39 d.
Bajardi, et al. (2011) (*11*)	• SEIR-like Global Epidemic and Mobility model of using	• 40 or 90% international travel	• Early stage of the outbreak	• Estimated mean delay of the arrival of infection in influenza-

International travel restriction	Study setting	Specific measures (Location and methods)	Timing of implementation	Study results by effective indicators
	H1N1 Pandemic influenza data 2009 (R_0 = 1.7 with generation interval set 3.6 d)	restriction to and from Mexico		unaffected countries was less than 3 d (40% restriction), and ≈2 weeks (90% restriction).
Brownstein, et al. (2006) (*12*)	• Seasonal influenza in U.S.<br>• R_0 = Not available<br>• Time series analysis<br>• Weekly influenza mortality for nine influenza seasons (from 1996–1997 to 2004–2005)	• International flight volume decreased by 27%	• Since September 11, 2001	• The peak of influenza mortality was delayed by 2 weeks.
Chong, et al. (2012) (*13*)	• Stochastic SEIR model of Influenza H1N1 pdm (R_0 = 1.4) in Hong Kong was conducted.<br>• The interval between the day of first infected cases passage and 100 infected cases passage time at the border was measured by different level and mode of border travel restriction and compared with no restriction	• 90, or 99% travel restriction between Hong Kong and mainland China on different modes of transportation including air, land, and sea	• On the day following the first global case identified	• A 99% international air travel restriction may delay the interval between the first imported case and one hundred infected case passed the border by a week and the epidemic peak delayed by two weeks.<br>• A 99% international travel restriction of both air and land travel may delay the interval (passage time) by an additional one to two weeks, and the epidemic peak may be delayed ≈3.5 weeks.<br>• A 99% restriction of all modes of transportation may delay the interval by an additional 2 mo, and delay the epidemic peak time by ≈12 weeks.
Cooper, et al. (2006) (*14*)	• Metapopulational model of SEIR (R_0 = 1.8–5) in Hong Kong, London, Sydney	• 50, 90, 99, or 99.9% international air travel restriction from affected cities with susceptibility of 10%, 60%, or 100%.	• After 1000 infected cases occurred in the originating city (Hong Kong, London and Sydney) or 100 cases occurred in other cities.	• Median epidemic peak delay can be ranged between 7 and 102 d.
Eichner, et al. (2009) (*15*)	• Probabilistic model using a travel data from Pacific Island Countries and Territories (R_0 = 1.5, 2.25, and 3.0).<br>• Using these data, expected number of infected case entering the island was calculated, and the probability that an island either suffer an epidemic or escape a pandemic was assessed.	• 79 or 99% restriction	• Very beginning of a pandemic until the end or the failure to prevent case-introduction	• Among 17 pacific island countries and territories, with 99% travel restriction, six countries (with R_0 = 1.5) and four to five countries (with R_0≥2.25) would be likely escape the pandemic influenza with more than 50% probability.<br>• However, with 79% travel restriction, only one country (with R_0 = 1.5) and no country (with R_0≥2.25) was likely to escape the pandemic.
Epstein, et al. (2007) (*16*)	• Stochastic global NSSEIR model of pandemic influenza (R_0 = 1.4, 1.7, or 2.0) was performed.<br>• The interval was measured between the outbreaks occurring in Hong Kong, London, or Sydney and the case-passage time to U.S.	• 90, 95, or 99% travel restriction	• Implemented when reached threshold of 1000 cumulative infectious cases	• First passage time may delay from 18 d to 31 d (outbreak originated from Hong Kong), from 7days to 27 d (from Sydney), and no delay (from London) with R_0 = 1.7.<br>• The delays are larger for smaller R_0.
Ferguson, et al. (2006) (*17*)	• Simulation using stochastic spatially structured mathematical individual-based model of SEIR using the scenarios of pandemic influenza (R_0 = 1.4–2.0) in U.S.	• 90, 99, or 99.9% restriction to U.S.	• From 30 d of global pandemic	• Imported infections might delay the epidemic peak of the U.S. by 1.5 (90% restriction), 3 (99%), or 6 (99.9%) weeks

International travel restriction	Study setting	Specific measures (Location and methods)	Timing of implementation	Study results by effective indicators
Hollingsworth, et al. (2006) (*18*)	• Simple mathematical SEIR model of an epidemic in a source country with asymptomatic cases exported to any of 100 other countries (R_0 = 1.8)<br>• Assumed 100 cases per day occurred	• 80, 90, or 99% travel restriction	• On the 20th day from first case occurred	• The mean time delay exporting the infected case is 5.3 (80% of restriction), 11.7 (90%), and 131.7 d (99%)
Lam, et al. (2011) (*19*)	• Simple stochastic model on heterogeneously mixing population on the H1N1–2009 influenza pandemic in Hong Kong (R_0 = 1.2, 1.6, or 2.0)<br>• Daily average of 10 imported cases for 50 d	• 100% travel restriction among children	• After pandemic declared	• Children-selective travel restriction may delay an epidemic for 19–35 d (R_0 = 1.2), and less than 15 d (R_0 = 1.6 and 2.0)

SEIR: Susceptible, exposed, infectious, and recovered.
NSSEIR: Nonsusceptible, susceptible, exposed, infectious and recovered.

3. Border closure

3.1 Terminology

The border closure is complete prevention of movement of individuals into and out of particular country (*9*).

3.2 Search strategy

Literature searches were conducted on the databases including PubMed, Medline, EMBASE and Cochrane Library for articles published from 1946 to 28 April 2019 using search terms shown in Appendix Table 5. Inclusion criteria were primary research evaluating border closure for influenza pandemics in the community setting. Studies had to demonstrate any effectiveness following border closure in the community. We excluded studies conducted in healthcare settings, animal-related studies, systematic reviews and/or meta-analysis without primary data, not measuring effectiveness of border closure to the community, and article type of letter, commentary or news without primary data. Two reviewers (SR and HG) contributed to the title, abstract, and full-text screening (Appendix Table 5).

Appendix Table 5. Search strategy for border closure

Search terms	Search date	Reviewers
#1. travel or traveler or travelers or traveller or travelers<br>#2. border<br>#3. restrict or restriction or prohibit or prohibition or limit or limitation or control or closure<br>#4. influenza or flu<br>#5. #1 and #2 and #3 and #4	29 April 2019	SR<br>HG

3.3 Findings

82 records were identified and included in the title and abstract screening, and 71 were excluded. Eleven full-texts were assessed for eligibility and 1 full-length articles were identified for inclusion in the systematic review. The flowchart of study selection is shown in Appendix Figure 3 and the summary of the studies shown in Appendix Table 6.

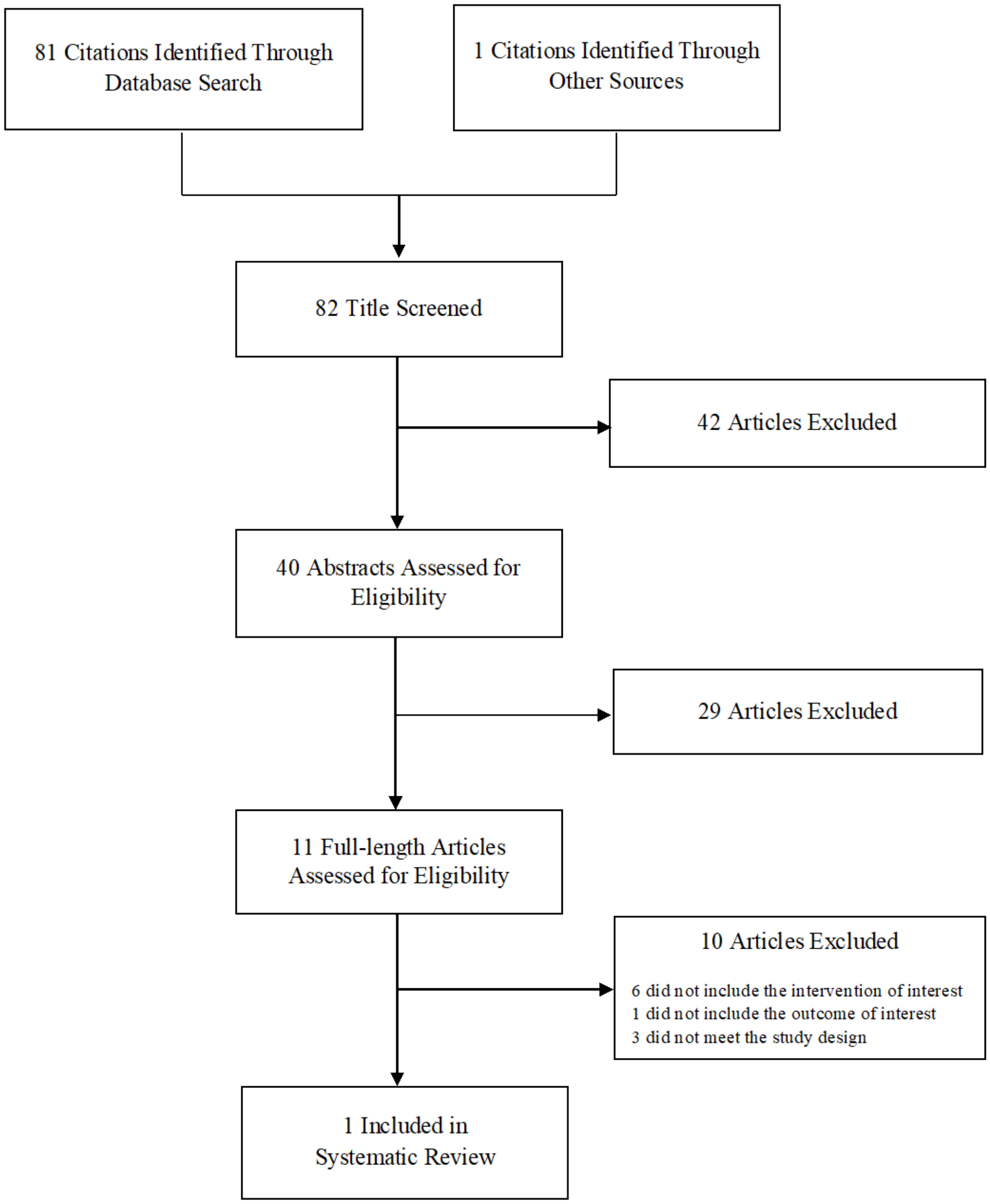

Appendix Figure 3. Flowchart of literature search and study selection for border closure.

Appendix Table 6. Summary of studies included in the review of border closure

Border closure	Study setting	Specific measures (Location and methods)	Timing of implementation	Study results by effective indicators
McLeod, et al (2008) (*20*)	• Reviewed mortality data and arrival of pandemic for 11 South Pacific Island jurisdictions in the 1918/1919 influenza pandemic	• 5–7 d' maritime quarantine<br>- American soma (5 d)<br>- Australia, Tasmania, New Caledonia (7 d)	• American Samoa in November 23, 1918, Australia in October 1918, and Tasmania January 27, 1919 implemented border control measures.	• Arrival of pandemic was significantly delayed and death rates attributed to influenza per 1000 population reduced compare with the other Pacific Island Jurisdiction<br>- American Samoa (arrival of pandemic; 1920, no death reported)<br>- Australia (early January 1919, 2.4 death reported)<br>- Tasmania (August 1919, 0.81 deaths reported)<br>- New Caledonia (1921, less than 11 deaths reported)

References

1. Read JM, Diggle PJ, Chirombo J, Solomon T, Baylis M. Effectiveness of screening for Ebola at airports. Lancet. 2015;385:23–4. PubMed https://doi.org/10.1016/S0140-6736(14)61894-8

2. European Centre for Disease Prevention and Control. Infection prevention and control measures for Ebola virus disease: entry and exit screening measures. 2014 [cited 2019 Jul 10]. https://www.ecdc.europa.eu/en/publications-data/infection-prevention-and-control-measures-ebola-virus-disease-entry-and-exit

3. Caley P, Becker NG, Philp DJ. The waiting time for inter-country spread of pandemic influenza. PLoS One. 2007;2:e143. PubMed https://doi.org/10.1371/journal.pone.0000143

4. Cowling BJ, Lau LL, Wu P, Wong HW, Fang VJ, Riley S, et al. Entry screening to delay local transmission of 2009 pandemic influenza A (H1N1). BMC Infect Dis. 2010;10:82. PubMed https://doi.org/10.1186/1471-2334-10-82

5. Malone JD, Brigantic R, Muller GA, Gadgil A, Delp W, McMahon BH, et al. U.S. airport entry screening in response to pandemic influenza: modeling and analysis. Travel Med Infect Dis. 2009;7:181–91. PubMed https://doi.org/10.1016/j.tmaid.2009.02.006

6. Yu H, Cauchemez S, Donnelly CA, Zhou L, Feng L, Xiang N, et al. Transmission dynamics, border entry screening, and school holidays during the 2009 influenza A (H1N1) pandemic, China. Emerg Infect Dis. 2012;18:758–66. PubMed https://doi.org/10.3201/eid1805.110356

7. Fang LQ, Wang LP, de Vlas SJ, Liang S, Tong SL, Li YL, et al. Distribution and risk factors of 2009 pandemic influenza A (H1N1) in mainland China. Am J Epidemiol. 2012;175:890–7. PubMed https://doi.org/10.1093/aje/kwr411

8. Wood JG, Zamani N, MacIntyre CR, Beckert NG. Effects of internal border control on spread of pandemic influenza. Emerg Infect Dis. 2007;13:1038–45. PubMed https://doi.org/10.3201/eid1307.060740

9. US Homeland Security Council. National strategy for pandemic influenza implementation plan. 2006 [cited 2019 Jul 10]. https://www.cdc.gov/flu/pandemic-resources/pdf/pandemic-influenza-implementation.pdf

10. Ciofi degli Atti ML, Merler S, Rizzo C, Ajelli M, Massari M, Manfredi P, et al. Mitigation measures for pandemic influenza in Italy: an individual based model considering different scenarios. PLoS One. 2008;3:e1790. PubMed https://doi.org/10.1371/journal.pone.0001790

11. Bajardi P, Poletto C, Ramasco JJ, Tizzoni M, Colizza V, Vespignani A. Human mobility networks, travel restrictions, and the global spread of 2009 H1N1 pandemic. PLoS One. 2011;6:e16591. PubMed https://doi.org/10.1371/journal.pone.0016591

12. Brownstein JS, Wolfe CJ, Mandl KD. Empirical evidence for the effect of airline travel on inter-regional influenza spread in the United States. PLoS Med. 2006;3:e401. PubMed https://doi.org/10.1371/journal.pmed.0030401

13. Chong KC, Ying Zee BC. Modeling the impact of air, sea, and land travel restrictions supplemented by other interventions on the emergence of a new influenza pandemic virus. BMC Infect Dis. 2012;12:309. PubMed https://doi.org/10.1186/1471-2334-12-309

14. Cooper BS, Pitman RJ, Edmunds WJ, Gay NJ. Delaying the international spread of pandemic influenza. PLoS Med. 2006;3:e212. PubMed https://doi.org/10.1371/journal.pmed.0030212

15. Eichner M, Schwehm M, Wilson N, Baker MG. Small islands and pandemic influenza: potential benefits and limitations of travel volume reduction as a border control measure. BMC Infect Dis. 2009;9:160. PubMed https://doi.org/10.1186/1471-2334-9-160

16. Epstein JM, Goedecke DM, Yu F, Morris RJ, Wagener DK, Bobashev GV. Controlling pandemic flu: the value of international air travel restrictions. PLoS One. 2007;2:e401. PubMed https://doi.org/10.1371/journal.pone.0000401

17. Ferguson NM, Cummings DA, Fraser C, Cajka JC, Cooley PC, Burke DS. Strategies for mitigating an influenza pandemic. Nature. 2006;442:448–52. PubMed https://doi.org/10.1038/nature04795

18. Hollingsworth TD, Ferguson NM, Anderson RM. Will travel restrictions control the international spread of pandemic influenza? Nat Med. 2006;12:497–9. PubMed https://doi.org/10.1038/nm0506-497

19. Lam EH, Cowling BJ, Cook AR, Wong JY, Lau MS, Nishiura H. The feasibility of age-specific travel restrictions during influenza pandemics. Theor Biol Med Model. 2011;8:44. PubMed https://doi.org/10.1186/1742-4682-8-44

20. McLeod MA, Baker M, Wilson N, Kelly H, Kiedrzynski T, Kool JL. Protective effect of maritime quarantine in South Pacific jurisdictions, 1918–19 influenza pandemic. Emerg Infect Dis. 2008;14:468–70. PubMed https://doi.org/10.3201/eid1403.070927
